# *“I’ve made this my lifestyle now”*: a prospective qualitative study of motivation for lifestyle change among people with newly diagnosed type two diabetes mellitus

**DOI:** 10.1186/s12889-018-5114-5

**Published:** 2018-01-31

**Authors:** Simon J. Sebire, Zoi Toumpakari, Katrina M. Turner, Ashley R. Cooper, Angie S. Page, Alice Malpass, Robert C. Andrews

**Affiliations:** 1 Centre for Exercise, Nutrition & Health Sciences, School for Policy Studies, University of Bristol, 8 Priory Road, Bristol, BS8 1TZ UK; 2 Department of Population Health Sciences, Bristol Medical School, University of Bristol, Bristol, UK; 3 The National Institute for Health Research Collaboration for Leadership in Applied Health Research and Care West (NIHR CLAHRC West) at University Hospitals Bristol NHS Foundation Trust, Bristol, UK; 4 National Institute for Health Research Bristol Biomedical Research Centre, University Hospitals Bristol NHS Foundation Trust and University of Bristol, Bristol, UK; 5 Centre for Academic Primary Care, Bristol Medical School, University of Bristol, Bristol, UK; 6 Institute of Biomedical and Clinical Sciences, University of Exeter Medical School, Medical Research, RILD Level 3, Barrack Road, Exeter, Devon EX2 5DW UK

**Keywords:** Type 2 diabetes, Motivation, Behaviour change, Intervention, Qualitative

## Abstract

**Background:**

Diagnosis with Type 2 Diabetes is an opportunity for individuals to change their physical activity and dietary behaviours. Diabetes treatment guidelines recommend theory-based, patient-centred care and advocate the provision of support for patient motivation but the motivational experiences of people newly diagnosed with diabetes have not been well studied. Framed in self-determination theory, this study aimed to qualitatively explore how this patient group articulate and experience different types of motivation when attempting lifestyle change.

**Methods:**

A secondary analysis of semi-structured interview data collected with 30 (n female = 18, n male = 12) adults who had been newly diagnosed with type two diabetes and were participants in the Early ACTID trial was undertaken. Deductive directed content analysis was performed using NVivo V10 and researcher triangulation to identify and describe patient experiences and narratives that reflected the motivation types outlined in self-determination theory and if/how these changed over time.

**Results:**

The findings revealed the diversity in motivation quality both between and within individuals over time and that patients with newly-diagnosed diabetes have multifaceted often competing motivations for lifestyle behaviour change. Applying self-determination theory, we identified that many participants reported relatively dominant controlled motivation to comply with lifestyle recommendations, avoid their non-compliance being “found out” or supress guilt following lapses in behaviour change attempts. Such narratives were accompanied by experiences of frustrating slow behaviour change progress. More autonomous motivation was expressed as something often achieved over time and reflected goals to improve health, quality of life or family time. Motivational *internalisation* was evident and some participants had integrated their behaviour change to a new way of life which they found resilient to common barriers.

**Conclusions:**

Motivation for lifestyle change following diagnosis with type two diabetes is complex and can be relatively low in self-determination. To achieve the patient empowerment aspirations of current national health care plans, intervention developers, and clinicians would do well to consider the *quality* not just quantity of their patients’ motivation.

**Trial registration:**

ISRCTN ISRCTN92162869. Retrospectively registered

## Background

Patient empowerment is a cornerstone of contemporary medicine and is central to national health care plans [1]. Individuals are increasingly encouraged, with support from professionals, to manage their own health. For this approach to be effective, a detailed understanding of how patients experience regulating their behaviour when they initiate and attempt to sustain health behaviour change is needed.

Approximately 6% of the adult population in England have diabetes, 90% are cases of Type 2 Diabetes Mellitus (T2DM) and the prevalence of T2DM is rising [2]. The burden of T2DM on individuals’ health (i.e., increased risk of cardiovascular disease, amputation, kidney disease, retinopathy and depression) and the economy are well documented and preventing, managing and treating diabetes are public health priorities [2].

The point of diagnosis with T2DM is an opportunity for clinicians to help patients initiate changes in lifestyle behaviours such as physical activity and diet [3]. Guidelines for the care of adults with T2DM build on a foundation of patient-centred care, and advocate the provision of theory-based patient education at, or soon after diagnosis to create personalised management plans, combining advice on diet, increasing physical activity and losing weight [3]. It is also suggested that patients try to improve their diet and increase their physical activity for 3 months before starting medication [4]. Related guidance (e.g., National Institute for Health and Care Excellence) on changing lifestyle behaviours such as physical activity suggests a range of techniques to *motivate and support* individual-level change including helping patients understand the consequences of their health-related behaviour, goal setting, and devising coping strategies to prevent relapse [5]. However, despite this potentially complementary dual focus on patient self-regulation and motivation, current guidelines do not consider the degree to which patients’ motivation for lifestyle change itself is or is not self-regulated.

Recently Fisher et al. [6] have stated that the efficacy of diabetes-directed interventions “is often dependent upon on how well a clinician is able to support personal engagement and motivation of the person with diabetes to use these new tools and knowledge consistently, and as directed”. Self-determination theory (SDT) [7] is a psychological framework of motivational self-regulation that has been extensively applied to physical activity [8], diet [9], medication adherence [10] and diabetes control interventions [11–13]. Rather than considering only the *quantity of people’s motivation* (i.e., motivated vs. not motivated) as in previous work with patients with T2DM [14], within SDT, the *quality* of motivation is considered based on the extent to which it is self-regulated [15].

Within SDT (Table 1), the most self-determined form of motivation is *intrinsic motivation*, where behaviour is driven by interest, enjoyment or the satisfaction that it brings. Types of motivation that are not intrinsic but based on tangible consequences or outcomes can vary in their level of autonomy/self-regulation. The most autonomous form (*integrated motivation*) is where motivation is derived from an alignment of the outcomes of a given behaviour (e.g., healthy eating) with a person’s broader sense of self, values or goals. Less self-determined, but still considered autonomous, is *identified motivation* which is based on personally important or valued benefits of an activity (e.g., valued health or social benefits of being active). *Introjected motivation* is a form of controlled motivation where self-imposed sanctions such as avoiding guilt or gaining contingent self-esteem drive behaviour whereas *external motivation* represents motivation based on a desire to comply with external demands or requests, avoid punishments or to gain rewards. Finally, *amotivation represents* an absence of motivation or intention to act. The dynamic process (although not necessarily linear) through which individuals’ progress from less to more self-regulation/autonomous motivation is called *internalisation* [15].

**Table 1:** Types of motivation along the Self-determination Theory continuum and diet/physical activity examples

	Amotivation	Extrinsic Motivation				Intrinsic motivation
		*Controlled regulations*		*Autonomous regulation*		
	Non-regulation	External Regulation	Introjected Regulation	Identified Regulation	Integrated Regulation	Intrinsic Regulation
Motivation type description	Lack of motivation or intention to act	Lifestyle behaviour change is to avoid punishment or gain a reward	Lifestyle change aims at avoiding guilt or enhancing self-worth	Lifestyle changes are personally important or valued	Lifestyle behaviours are in harmony with other personal values and goals	Lifestyle behaviours are enjoyable or inherently satisfying to do
Diet / physical activity example	Not changing one’s lifestyle behaviours or passively *going through the motions*	Eating less confectionary to avoid being *told off* by a dietician	Exercising because one feels they *should,* and will feel guilty if one doesn’t	Maintaining one’s physical fitness is a personally important goal	Eating a healthily is consistent with one’s goals to be physically active	Trying out new healthy recipes is satisfying and fun

Research amongst people with T2DM has shown that autonomous motivation is positively associated with lifestyle behaviours such as physical activity [16], dietary self-care [17, 18], medication adherence [13], key mediators of behaviour change (e.g., action planning) [18] and sustained improvement in physical health including diabetes control [12]. There is also some evidence that controlled motivation (e.g., pressure to comply with advice or change ones behaviour to please others or to suppress feelings of guilt) is associated with improved dietary self-care amongst people with newly-diagnosed T2DM [19]. Together, this evidence highlights the beneficial outcomes associated with autonomous motivation but also that controlled motivation may play a role in the lifestyle changes of people with newly diagnosed diabetes. This is not surprising given that upon diagnosis, patients commonly receive information (e.g., identifying previous lifestyle behaviours that may have contributed to diabetes, possible future health complications, the lifestyle change needed to manage their symptoms, and weight and blood glucose targets to meet); interactions that have the potential to trigger either autonomous (e.g., identifying personally important reasons for change) or controlled (e.g., feeling guilty or pressured) motivation for change.

The majority of previous research has studied motivation amongst people with T2DM using quantitative questionnaires and existing qualitative research has only identified motivational factors such as weight management and physical and mental well-being as motivating physical activity among people at risk of diabetes [20]. Despite calls from researchers [21], the *quality* of the motivation of people who are in early phases of initiating behaviour change following a diagnosis of diabetes has not been studied from the patients’ perspective. It is important to address this gap because understanding people’s motivational experiences at critical times of behaviour change can inform the design of patient-centred lifestyle interventions or care.

The present study aimed to: (1) qualitatively explore how people newly diagnosed with T2DM articulate and experience motivation for lifestyle change as proposed in SDT, and (2) to examine qualitative evidence for patients’ motivational internalisation over time (i.e., transition from controlled to autonomous motivation).

## Methods

### Study design

A secondary analysis of interview data collected with individuals who had been newly diagnosed with T2DM and were participants in the Early ACTID (Early ACTivity In Diabetes) trial.

### The early ACTID trial

Early ACTID was a lifestyle RCT, conducted between December 2005 and September 2009, at three sites in South West England, involving 593 adults aged between 30 and 80 years old who had received a diagnosis of T2DM within the previous 6 months [22]. Patients were recruited through GP practices and randomised to three arms; usual care (UC), intensive dietary advice (ID), or an intensive dietary advice and physical activity intervention (DPAI). Usual care comprised the provision of standard advice from a dietician at a baseline appointment, followed by two visits to a doctor blinded to treatment allocation at 6 and 12 months post-randomisation. The ID arm comprised UC plus 15 20-min appointments with a nurse or dietician for 12 months following randomisation where patients were encouraged to achieve a daily intake reduction of 500 Kcal and 5 to 10% loss of initial weight over 12-months. In addition to receiving UC and the ID intervention, patients in the DPAI arm were encouraged to increase their physical activity to at least 30 min of brisk walking on 5 days/week. The ID and DPAI interventions were not based on SDT or other psychological theory, however behaviour change techniques included; information provision, setting and negotiating achievable physical activity and/or eating behaviour and weight loss goals, weighing at appointments, barrier identification and assistance to overcome them, encouragement to self-monitor weight, completion of food and or/physical activity diaries and wearing a pedometer.

### Interviewee recruitment

At their baseline appointment, each participant was given information about the qualitative study and asked to consent to being approached to take part in an interview. Consenting individuals were purposefully sampled to ensure interviews were held with participants in each trial arm, with men and women of varying ages, and from the different trial sites. No relationship was established with participants before interview.

### Data collection

The aim of the original ACTID qualitative study was to explore how patients recently diagnosed with T2DM experience and manage their condition as well as monitoring implementation and identifying improvements to the intervention. Thirty patients (n female = 18) were interviewed, comprising 6 from the UC arm (n female = 3), 12 from the ID arm (n female = 8) and 12 (n female = 7) from the DPAI arm. Participants were aged between 40 and 72 years. Semi-structured interviews were conducted (by AM) at 6-months (face-to-face interview) and 9-months (telephone interview) post-randomisation. The 6-month interview was timed so as not to influence participants’ experience of the initial intervention stages. By 9-months participants had received the majority of intervention and sufficient time to make lifestyle changes following diagnosis and inclusion in the trial.

The 6 month interview covered topics including response to diagnosis, use of the information, behavioural changes and relationship with the clinical team [23]. The 9 month interview covered topics including diet and/or exercise changes, maintaining change, barriers, coping with reduce appointment frequency and the trial ending. Neither the interview guide nor the interviews themselves were based on SDT, although the topic of motivation for change was discussed in depth. Within the original data collection team, KM and AM discussed themes and data saturation pertaining to the research questions. At the time, this was felt to have been reached. The 6 month (*n* = 30) and 9 month (*n* = 29, one participant did not participate in a 9 month interview) interviews were, on average, 90 and 15 min in duration respectively. Interview audio recordings were transcribed verbatim. Ethical approval was given by the Bath Research Ethics Committee (05/Q2001/5).

### Data analysis

The analysis combined both supra (i.e., by answering a new theoretical question) and supplementary (i.e., a more in depth investigation of motivation themes which were not addressed fully in the primary study) secondary qualitative analysis [24]. Initial analysis involving three of the authors indicated that there were sufficient accounts to explore the quality of participants’ motivation. All available data were analysed using directed content analysis which is appropriate where an existing theory can guide research questions and initial theory-based themes and can be supported, challenged or extended [25]. Analysis was primarily deductive and sought to identify patient experiences and narratives that reflected the motivation types outlined in Table 1. Further, a within-participant analysis probed for evidence of motivational internalisation between the 6 and 9 month interviews. Alongside the deductive analysis, coding was flexible to allow participants’ narratives and personal context to guide the complexity of themes and new themes to emerge.

Transcripts were loaded to NVivo software (QSR International Pty Ltd. Version 10, 2012) to enable coding of the data and to facilitate the organisation of codes into themes and subthemes. Coding was undertaken by two researchers (ZT & SJS) who had experience of studying SDT. Both began by coding the same three transcripts, discussing their initial interpretations/codes and then refining the initial coding frame (i.e., new codes being added and existing codes being deleted/refined). The coding frame was then applied to another five transcripts, further refined and agreed and then applied to the remaining 52 transcripts which were divided equally between ZT and SJS. Researcher triangulation was undertaken via independent coding of transcripts and regular meetings to discuss code refinements and emergent themes. The researchers agreed that the quotes presented were representative of the themes.

## Results

The results are presented as six themes reflecting the motivation types in SDT (See Table 1) with narratives reflecting internalisation (or the lack of) integrated alongside each motivation type. Participant gender, trial arm allocation (ID: Intensive Dietary Advice, DPAI: Intensive Dietary Advice & Physical Activity, & UC: Usual Care) and interview time point are shown for each quote.

### Amotivation

Some participants were reluctant to change and articulated a passivity towards any changes they reported. Ignoring one’s diabetes, feeling helpless, not able to change or resigned to one’s current way of life and not believing the health benefits of recommended treatments were also common:

*… the other tablets I take is all in the morning, this one is at night, then one night I forget, then the next night I forget and I can’t be bothered taking these because I don’t think - I can’t believe tablets would do [me any good] - you know?* (Mo, male, DPAI, 6 months)

Some participants felt helpless: “*At 66 I'm hardly … going to change very much” (Ronny, male, ID, 6 months)* and others reported acting against advice that was not in line with a “*good life*”, deceiving their health practitioners to appear compliant:

*Well they don't know I'm not doing anything, I'm living a good life and being a good boy. [laughs] I don’t tell ‘em, I'm back on scones, they just say “how’s your diet?” And I say “okay”.* (James, male, UC, 6 months)

Three months later, James had not changed his motivation or diet:

*Well I never hardly made any [diet changes], only cut out me scones and Rich Tea biscuits, that’s all I’ve done, but that’s gone by the board [been abandoned] the last few months, mind.* (James, male, UC, 9 months)

### External motivation

The majority of the participants referred to their motivation being controlled by external sources when first changing behaviour post-diagnosis. Participants felt restricted in what they were “*allowed to eat*” (Frank, male, UC, 6 months) or the exercise that they “*have to*” do (Nina, female, DPAI, 6 months), found the changes unenjoyable, and a threat to their quality of life. External motivation was commonly interpreted from narratives about patient *compliance* with the Early ACTID health practitioners’ recommendations or goals to avoid negative outcomes (such as increasing medication).

*I can use those [books] for reference for restricting carbohydrate intake, which I'm trying to do at the moment. It’s the latest aim of my - or goal of my dietician. And that way I can hopefully reduce these glucose levels a bit more … I don’t want to get a third tablet a day, that's what I'm trying to avoid.* (Stuart, Male, ID, 6 months)

Some participants’ behaviours were motivated by wanting to demonstrate that they had been “*behaving*” between appointments, completing behaviour change activities such as diet diaries, and a fear that non-compliance would be identified by measurements:

*Knowing that I'm going to see [practitioner] every so often … part of me says “well you’ve got to behave yourself girl because you're going to be going and seeing them, and they’ll know if you haven't been behaving yourself, your weight and your levels and everything else”* (Wendy, Female, ID, 9 months)

Narratives rich in external motivation coincided with reports of challenges to behaviour change and disappointment when effort was not rewarded with the desired outcomes such as weight loss:

*So we’re there cutting, cutting, cutting, and doing everything they say and the weight doesn’t come off, ‘oh you’ve got to get your output up’, so my output is now up, the weight is not coming off, you know?* (Hugh, Male, DPAI, 6 months)

The majority of participants who articulated external motivation at 6 months, appeared to remain controlled by external sources 3 months later. During their second interview their low enjoyment of lifestyle changes persisted, and they reported challenges, lapses and slow progress towards change that they did not feel in control of:

*Things have just gone on like a slow train without any stations*. (Rose, Female, DPAI, 9 months)

Participants (in all trial arms) were commonly concerned about losing the professional support when the intervention ended that provided them with motivation they needed to sustain change.

*Through not seeing [practitioner] so much, no one is on top of me, keeping … I am like a little boy really, I suppose, I need somebody to give me a kick up the backside … and I know it’s coming to an end now as well, so I'm getting a little bit lax I think.* (Mo, male, DPAI, 9 months)

### Introjected motivation

Motivation based on personal pressures such as avoiding guilt were commonly rooted in partially-internalised lifestyle advice. Participants’ understood the reasons for lifestyle change but appeared relatively controlled by these reasons rather than describing them as personally valued. Participants commonly reported introjected exercise motivation to burn calories consumed during dietary lapses:

*Admittedly if I've eaten something that I know I shouldn’t have and I haven’t yet exercised, I'm more determined to go and exercise then, because … I've got to get rid of that, I've got to burn it off.* (Wendy, Female, ID, 9 months)

Similarly, introjected motivation was common where participants had hung their self-worth on successful behaviour change but not achieved it. They judged their behaviour as “*wrong*” versus right and themselves as “*naughty*”, “*stupid*” or at “*fault*”.

*I'm going to be good and go back on my diet, it’s entirely my fault because I know the rights and the wrongs and the dos and the don’t dos … it was also summer and barbeques and I just think I’d been very naughty and stupid and allowed this to happen, but I do see the dietician on Thursday, so it is entirely my own fault*. (Diana, female, DPAI, 9 months)

Showing the interplay between external and introjected motivation over time, at 6 months Clive (Male, DPAI) was motivated by the reward of preventing a worsening of his diabetes but viewed certain dietary changes as punishment:

*I'm very motivated by rewards if you like, and I consider the reward for not doing that is my diabetes not getting any worse, and if I can hold it at that, then I’ll punish myself with other things, such as giving up sugars and sweets* (Clive, Male, DPAI, 6 months)

Three months later, Clive’s diabetes had worsened, he had been prescribed medication to improve his blood sugar control and felt guilty for not having made changes sooner. He now felt internal pressure to adopt the lifestyle advice “*more seriously*”.

*I was cursing myself for not giving it [unhealthy food] all up and trying to keep on the diet rather than have to go onto tablets … I’ve still left myself subject to eat some sweet things a week, and had I known that it would take me out of the diet side onto tablets I would have made even more effort I think … So yeah, I really must settle down with these tablets - and take it a little bit more seriously than I have done.* (Clive, Male, DPAI, 9 months)

### Identified motivation

At diagnosis, all participants had been informed of the potential health implications of diabetes and the benefits of lifestyle changes for glucose control. It was therefore unsurprising that many referred to valuing the experienced or anticipated health benefits of diet or physical activity changes including feeling fitter, healthier, improved quality of life and well-being. Others identified with maintaining their health in order to spend time with family and/or fulfil caring responsibilities:

*(I) don’t want to go blind, I don’t want my legs chopped off and I’d like to live a bit longer. [laughs] And I've got a family to look after. It’s all that sort of stuff. So I want to stay as healthy as I can for as long as I can, preferably with as little medication as I can.* (Pete, Male, DPAI, 6 months)

Like Pete, many participants reported identified motivation based on the importance they placed on avoiding deleterious health consequences of diabetes. While this form of motivation was initially an external motivation, and accompanied for some by fear of the consequences of not changing, many participants had internalised the health threat to become a personally valued outcome of change. Commonly, these motivations were based on participants wanting to avoid the ill health experienced by their relatives or friends:

*Knowing how my mother went with her diabetes and the fact that before she died she could only just about see, I thought “well I don’t want that happening to me”.* (Monica, female, UC, 6 months)

Further, the personal importance placed on avoiding ill health was accompanied by participants taking responsibility for change, following advice and wanting to avoid future feelings of shame associated with *not* changing:

*I don’t want it [heart problems] to happen to me because I'm not following recommended lines. If it happened to me for any other reason then that’s something that I probably can’t control, but if there is something that I can contribute to stop it happening then I will do so.* (Robert, male, UC, 6 months)

For some participants, their diagnosis with diabetes provided “*a stronger motivation that is bigger than the other motivations for not doing it”* (Alice, Female, ID, 6 months). This was in contrast to their previous, less effective appearance-based motivations for dieting:

*That's what I was like with my diets, I [would] not- stick to them, because - well you don’t think there's any health reason why you should stick to this thing apart from vanity with slimming. But as soon as you're told you're diabetic, it’s in your best interests to get that weight off and stick to a healthy diet. So I suppose in a roundabout way that complication helps you another way, and then I feel much better now for that.* (Mary, female, DPAI, 6 months)

Participants carried their identified motivation from 6 to 9 months but also expressed their need for continued collaborative professional or social support to help them monitor their diabetes and/or support their motivation:

*I am frightened of it [Early ACTID] ending. I would like to be monitored by experts, but I don’t think they are experts at my surgery … if I was monitored regularly like I am now, I would know that it was the diabetes that I allowed to get out of control and I’ll do something about it. Without knowing I'm very worried*. (Frank, Male, UC, 9 months)

### Integrated motivation

Some participants described how over time their lifestyle behaviours had become motivated by forming a new pattern, routine, or a way of life. Many of these statements were articulated at the 6 month interviews suggesting that some participants had internalised the motivation for lifestyle change relatively quickly after diagnosis:

*I think I’ve entered now into a pattern and a way of life* (Jill, female, DPAI, 6 months).

Many narratives like this reflected an internalisation process whereby participants’ motivation was initially controlled but over time aligned lifestyle recommendations with their perception of a good quality of life:

*Obviously it’s becoming a way of life now, what I'm doing, it’s becoming a way of life, and I don’t think about it so much. The first few months I did, I thought “I mustn’t do this, I mustn't do that”, and [practitioner] said “I think you're being a bit hard on yourself here, you're being a bit too strict and you won’t keep it up”. But now I'm settled into a very comfortable way of life with it.* (Nora, female, ID, 6 months)

References to behavioural habits, viewing exercise or healthy eating as a personal characteristic, and flexible resilience to challenges such as dietary lapses or bad weather were common alongside integrated motivation:

*I've made this my lifestyle now, my exercise, it’s just such a routine now that I get up, I have my breakfast and I go out, and I just do it really. If it’s raining I go, if it’s absolutely lashing I might wait and say “I’ll go this afternoon”, but very rarely do I miss.* (Penny, Female, ID, 9 months)

Personal characteristics that supported internalisation of lifestyle changes included listening to advice from practitioners, taking responsibility for change, being positive, and persistent. Participants also referred to making gradual changes, perceiving that the time was right for change and balancing their new and more established lifestyle components (e.g., social life)

*Systematically [it] becomes part of your life … I try in life not to make a hard life any harder, just try and make it easy by getting to grips with this, listening to what you're told and being positive about this.* (Nora, female, ID, 6 months)

### Intrinsic motivation

Several participants reported enjoying their new way of eating or the relaxation and satisfaction brought about by exercise:

*The exercise now has become so much a part of my life that I don’t think about it as the fact that I must do it as a chore, I do it because I enjoy it, I go out, the hour walking sorts the brain out for the day, its relaxing, sometimes I'm out and I'm back before I've gone, before I realise* [laughs]. *Yes, I enjoy it.* (Penny, Female, ID, 6 months)

Intrinsic motivation was sometimes supported by identified and introjected motivations at the moment of deciding to exercise:

*Some days I have to make myself do it, but I always feel better when I come back. I always feel I've got more energy, and more get up and go when I've been. It sounds daft don’t it- it’s almost like somebody strikes match and you do it and then you come back.* (Sylvia, female, DPAI, 6 months)

Robert demonstrated internalisation where his motivation transitioned from eating to follow recommendations to a combination of integrated (his eating “*style has changed*”) and intrinsic motivation (he now enjoys different foods). He thought that it was important to be motivated by enjoyment rather than pressure.

*When you see somebody alongside you with a meal that you like but you can’t have, I don’t find it envious anymore that they are eating something I would like to have eaten, my style has changed, I now enjoy a different type of food, but the important thing is I enjoy it, it’s not simply because I know that that’s what I've got to eat.* (Robert, male, UC, 6 months)

## Discussion

This study is the first qualitative examination of the types of motivation for lifestyle change articulated in SDT amongst people newly diagnosed with T2DM. As the participants were involved in a lifestyle intervention, it could be argued that almost all had some motivation to change, but despite this, the findings highlight the diversity in motivation *quality* both between and within participants. The prospective data facilitated an analysis of motivation transitions.

Diagnosis with T2DM provokes a range of emotional responses [26], close scrutiny of patients’ lifestyle, threats to people’s social and personal identity and the need to construct a new identity representations [27]. It is not surprising therefore that many participants’ motivation for change was controlled, or not self-regulated. External motivation for diet or physical activity change was experienced as participants complying with what they perceived to be restrictive dietary advice and through fear of non-compliance (i.e., “lapses”) being identified in appointments or assessments. On the SDT continuum (Table 1) external and introjected motivation are located adjacently and in the theory, motivation is viewed as dynamic rather than static [15]. This was supported in our findings as participants often experienced these motivations concurrently, by complying with recommendations and labelling themselves as “good” or “naughty” and their behaviour as “right” or “wrong” based on the extent to which their behaviour change was successful. Consistent with previous research with exercisers pursuing extrinsic (relatively controlling) goals [28], participants whose motivation was relatively controlled experienced frustrating slow progress towards rigidly defined end point goals (e.g., weight loss). These experiences of controlled motivation amongst people with newly diagnosed T2DM are a source of internal conflict and potential barriers to their development of self-regulation.

Previous work has shown that autonomous motivation is associated with physical activity and healthy eating [8, 9]. Identified motivation (i.e., personally important valuing of a behaviour) mainly stemmed from the value participants placed on health, quality of life and family responsibilities which they understood to be compromised by uncontrolled diabetes. Improved health is an intrinsic goal [29] which, relative to extrinsic goals, such as improved appearance, is associated with autonomous motivation and physical activity behaviour [30, 31]. The results add experiential support to this finding as for some participants health-based reasons for change, prompted by their T2DM diagnosis, were more motivating than their previous extrinsic appearance-based weight loss goals. Diabetes diagnosis may offer an opportunity to help individuals identify meaningful intrinsic goals (e.g., health or family time) which will likely underpin autonomous motivation.

Despite the participants being relatively newly diagnosed with T2DM some reported integrated motivation (i.e., physical activity or healthy eating being part of their identity), which plays an important role in motivating diet [9] and physical activity [32]. Having internalised early controlled motivations (i.e., moved from controlled to autonomous motivation), participants’ new lifestyle had become a pattern or a way of life which was robust to challenges. Integrated motivation developed over time and internalisation was supported by personal factors such as a positive attitude, resilience to barriers (e.g., bad weather), persistence and practitioners who encouraged gradual change.

Intrinsic motivation (i.e., being motivated by enjoyment, interest and satisfaction) was articulated least frequently, although some participants enjoyed their exercise and diet changes and this was commonly supported by integrated motivation. It is to be expected that new physical activity and eating behaviours may not yet be intrinsically motivated in a sample such as ours, and it is possible that for some patients, or for some behaviours (e.g., cutting down on high sugar foods) which patients find enjoyable, identified motivation for change (i.e., identifying a health-based value) may be a more realistic and adequate motivational target. Indeed it is suggested that maintenance of lifestyle behaviours, such as exercise, is most likely when a person has a combination of intrinsic, identified and integrated motivation types [33]. Collectively, the findings suggest that if T2DM patients can be supported to internalise their motivation to the point of identifying a personal benefit, or integrate changes as part of an enjoyable way of life, such changes may be more sustainable and resilient to common challenges to behaviour change (e.g., lack of time, periods of holiday, & changes in routine).

Recent quantitative research using SDT has sought to identify how different types of motivation for physical activity commonly cluster within individuals [21, 32, 34]. Amongst adults with T2DM, Gourlan et al. [21] identified a “self-determined” profile (high scores on autonomous motivation types), a “moderate” profile (all motivation types moderately endorsed), and a “high combined” profile (all motivation types strongly endorsed plus moderate amotivation). Our findings add further experiential evidence to support the existence of these multifaceted motivation profiles which commonly include both autonomous and controlled motivation. For example, amongst patients who reported largely identified and intrinsic motivation, low-level controlled motivation (often introjected) supported their maintenance of behaviour change at times. Together, the findings support calls for future research to take a theory-driven person- rather than variable-centred approach to understanding motivation [35] and indicate that mixed-methods approaches may be particularly illuminating.

Largely regardless of their dominant motivation, participants articulated a need for structure in their care, commonly through provision of expert guidance and support. However, the nature of the structure sought differed depending on participants’ motivation. Specifically, participants mainly motivated by autonomous reasons sought support for their ongoing self-regulation (with particular interest in ongoing assessment of health outcomes), whereas participants mainly motivated by controlled reasons sought more continuous provision of motivation (i.e., being pushed or prompted) by a practitioner, family member or friend with references to paternalistic perspectives (e.g., “*like a little boy*”). The provision of structure is a cornerstone of autonomy-supportive clinical/interpersonal interactions, which aim to facilitate patients’ autonomous motivation and competence [36]. This study highlights the importance of considering the long term provision of support/structure for people newly diagnosed with T2DM for two reasons; first, the transition from controlled to autonomous motivation can take time, and ongoing continuity in expert support can create a space to facilitate patients’ internalisation, and second, it is clear that even participants who were relatively self-regulated did not want to be left on their own, rather they wanted professional support to “*keep on track*”. Our findings therefore support the distinction drawn in SDT between the provision of autonomy-support (i.e., support for self-regulation) and independence (i.e., being left to fend for oneself).

The findings of this study suggest that to achieve the patient empowerment aspirations of current national health care plans [1], clinicians would do well to consider the quality not just quantity of their patients’ motivation. Research suggests that physicians may not know whether their T2DM patients are motivated to change or not and recommend the regular measurement of patient motivation [14]. While our findings support a greater focus on patient motivation, we would argue that considering the *quality* of motivation is of primary importance. Our findings complement a recent framework for supporting engagement and motivation for behaviour change in people with diabetes which draws on multiple patient-centred approaches including SDT [6]. This framework provides clinicians with a pragmatic, three-step approach to building a supportive clinician-patient relationship. Our findings support this work by documenting patient motivational experiences in line with the framework’s underpinning theory that clinicians will likely experience in conversations with patients about behaviour change. Previous work has identified how concepts from SDT could be integrated into medical training [37] which would help clinicians become attuned to patients’ motivation quality and support patients’ motivational needs.

### Strengths and limitations

The qualitative data provided a rich person-centred resource with which we were able to extend previous variable-centred quantitative literature. The sample size was relatively large and the initial interviews were extensive. The repeated interviews helped us hear personal experiences of the dynamic nature of behaviour change and motivation. Despite these strengths, the follow-up interviews were shorter (although the transcripts suggested that discussions were detailed) and although there can be strength in not basing interviews on theory, more theory-driven follow up interviews would have allowed a more in-depth analysis of motivation change. Finally, while we have reported our secondary analysis methods transparently and used researcher triangulation to agree our interpretations, due to the lapse between data collection and the analysis, it was not possible to use other strategies, such as member checking.

## Conclusions

Understanding T2DM patients’ motivation for lifestyle behaviour change is considered central to successful patient-centred care [6]. Combining qualitative methods with SDT, we have identified the diverse motivational experiences of people newly diagnosed with T2DM. Participants who reported relatively dominant controlled motivation experienced initial behaviour change but this was often accompanied by internal conflict, frustration and a need for continual external prompting. Participants’ reporting more autonomous motivation approached behaviour change with more flexibility had integrated it in to a new way of life and wanted ongoing support for their self-regulation. The findings highlight the importance of understanding the quality of motivation in this group and carefully considering the types of motivation that are targeted in lifestyle interventions for people with T2DM.

## Abbreviations

DPAI: Dietary advice and physical activity
HbA1c: Glycated haemoglobin (A1c)
ID: Intensive dietary advice
SDT: Self-determination theory
T2DM: Type 2 diabetes mellitus
UC: Usual care
